# Supplementary material for: Norspermidine Is Not a Self-Produced Trigger for Biofilm Disassembly
Source: Cell. 2014 Feb 13;156(4):844–54. doi: 10.1016/j.cell.2014.01.012 (PMC3969229; doi:10.1016/j.cell.2014.01.012)
Supplement: Document S1. Tables S2–S4 [file mmc2.pdf]

**Table S2. Strains of *B. subtilis* from This Study, Related to Experimental Procedures**

| Strain | Relevant genotype/Description | Source/Construction |
|---|---|---|
| MC1061 | *E. coli F'lacIQ lacZM15 Tn10* (*tet*) | *E. coli* Genetic Stock Center |
| NCIB3610 | prototroph | *Bacillus* Genetic Stock Center |
| NCIB3610-H | prototroph | Losick Laboratory |
| 168 | *trpC2* | *Bacillus* Genetic Stock Center |
| *yaaO* | *168 yaaO::erm* | (Sekowska et al. 1998) |
| *eps* | 3610-H *eps(A-O)::tet* | (Branda et al. 2006) |
| NRS2450 | 3610 *eps(A-O)::tet* | SPP1 *eps* → NCIB3610 |
| NRS3088 | 3610 *yaaO::erm* | SPP1 *yaaO* → NCIB3610 |
| NRS3089 | 3610 *speA*::*spc* | (Burrell et al. 2010) |
| NRS3971 | 3610 *speA::spc eps(A-O)::tet* | SPP1 NRS3089 → NRS2450 |
| NRS3991 | 168 + pNW1111=pMAD-Δ*speD* | pNW1111 →168 |
| NRS3992 | 168 + pNW1110=pMAD-Δ*gabT* | pNW1110 →168 |
| NRS3996 | 168 *amyE*::$P_{hy\text{-}spank}$*gabT-lacI* (*spc*) | pNW1114 →168 |
| NRS4005 | 3610 Δ*speD* | SPP1 NRS3991 → NCIB3610 |
| NRS4007 | 3610 Δ*gabT* | SPP1 NRS3992 → NCIB3610 |
| NRS4104 | 3610 Δ*gabT amyE*::$P_{hy\text{-}spank}$*gabT-lacI* (*spc*) | SPP1 NRS3996 → NRS4007 |

**Table S3. Plasmids Used in This Study, Related to Experimental Procedures**

| Plasmid | Description | Source |
|---|---|---|
| pUC19 | High copy number cloning vector | (Yanisch-Perron et al. 1985) |
| pMAD | In-frame markerless deletion plasmid | (Arnaud et al. 2004) |
| pDR111 | *B. subtilis* integration vector for IPTG-induced expression | (Britton et al. 2002) |
| pNW1106 | pUC19-Δ*speD* | This work |
| pNW1107 | pUC19-Δ*gabT* | This work |
| pNW1110 | pMAD-Δ*gabT* | This work |
| pNW1111 | pMAD-Δ*speD* | This work |
| pNW1114 | pDR111-*gabT* coding region | This work |

**Table S4. Oligonucleotide Primers Used in This Study, Related to Experimental Procedures**

| Primer | Sequence 5’-3’ | Use |
|---|---|---|
| **NSW1500** | GCATGGATCCTTCCAACAACAACAGGAGCG | *speD* deletion cloning |
| **NSW1501** | CGCAATTTACTTTATAGTACGTCGACAGTCATGGACCCCCC TTAAC | *speD* deletion cloning |
| **NSW1502** | GTTAAGGGGGGTCCATGACTGTCGACGTACTATAAAGTAA ATTGCG | *speD* deletion cloning |
| **NSW1503** | GCATAAGCTTAGATCTCGACTTCGTCAATCTTAGCC | *speD* deletion cloning |
| **NSW1508** | GCATCCCGGGCAGTCAACAGCTGCATGAGC | *gabT* deletion cloning |
| **NSW1509** | CTTTCCAATGATTAAGCTCGTCTAGAACTCATGTGAATATC CCCCT | *gabT* deletion cloning |
| **NSW1510** | AGGGGGATATTCACATGAGTTCTAGACGAGCTTAATCATTG GAAAG | *gabT* deletion cloning |
| **NSW1511** | GCATAAGCTTAGATCTTGCTTGTGAACACATTTCCG | *gabT* deletion cloning |
| **NSW1524** | GCATAAGCTTATACCGACAGGGGGATATTCACATGAGTCAA AC | *gabT* complementation |
| **NSW1525** | GCATGCATGCTTAAGCTCGCAGGCCCGCCTCCAAGATGC | *gabT* complementation |
