# Supplementary material for: Norspermidine Is Not a Self-Produced Trigger for Biofilm Disassembly
Source: Cell. 2014 Feb 13;156(4):844–54. doi: 10.1016/j.cell.2014.01.012 (PMC3969229; doi:10.1016/j.cell.2014.01.012)
Supplement: Document S2. Article plus Supplemental Information [file mmc3.pdf]

Cell

Matters Arising

Norspermidine Is Not a Self-Produced Trigger for Biofilm Disassembly

Laura Hobley,[1] Sok Ho Kim,[2] Yukari Maezato,[2] Susan Wyllie,[3] Alan H. Fairlamb,[3] Nicola R. Stanley-Wall,[1,*] and Anthony J. Michael[2,*]
[1]Division of Molecular Microbiology, College of Life Sciences, University of Dundee, Dundee DD15EH, UK
[2]Department of Pharmacology, University of Texas Southwestern Medical Center, Dallas, TX 75390, USA
[3]Division of Biological Chemistry and Drug Discovery, College of Life Sciences, University of Dundee, Dundee DD15EH, UK
*Correspondence: n.r.stanleywall@dundee.ac.uk (N.R.S.-W.), anthony.michael@utsouthwestern.edu (A.J.M.)
http://dx.doi.org/10.1016/j.cell.2014.01.012
This is an open-access article distributed under the terms of the Creative Commons Attribution License, which permits unrestricted use, distribution, and reproduction in any medium, provided the original author and source are credited.

SUMMARY

Formation of *Bacillus subtilis* biofilms, consisting of cells encapsulated within an extracellular matrix of exopolysaccharide and protein, requires the polyamine spermidine. A recent study reported that (1) related polyamine norspermidine is synthesized by *B. subtilis* using the equivalent of the *Vibrio cholerae* biosynthetic pathway, (2) exogenous norspermidine at 25 μM prevents *B. subtilis* biofilm formation, (3) endogenous norspermidine is present in biofilms at 50–80 μM, and (4) norspermidine prevents biofilm formation by condensing biofilm exopolysaccharide. In contrast, we find that, at concentrations up to 200 μM, exogenous norspermidine promotes biofilm formation. We find that norspermidine is absent in wild-type *B. subtilis* biofilms at all stages, and higher concentrations of exogenous norspermidine eventually inhibit planktonic growth and biofilm formation in an exopolysaccharide-independent manner. Moreover, orthologs of the *V. cholerae* norspermidine biosynthetic pathway are absent from *B. subtilis*, confirming that norspermidine is not physiologically relevant to biofilm function in this species.

INTRODUCTION

The ability of microbial cells to colonize a habitat is frequently dependent on biofilm formation, a process by which microbial cells form a sessile community encased in a self-produced extracellular matrix (Costerton et al., 1995; Davey and O'Toole, 2000; Flemming and Wingender, 2010). This extracellular matrix is typically composed of extracellular DNA, proteins, and exopolysaccharides that together function to adhere cells to a surface and provide protection from external physical and chemical stresses (Branda et al., 2005; Flemming and Wingender, 2010). To ensure correct deployment of these extracellular macromolecules in Gram-positive and Gram-negative bacterial species, biosynthesis is tightly regulated (Joo and Otto, 2012; Vlamakis et al., 2013). The Gram-positive bacterium *Bacillus subtilis* has emerged as a model organism for deciphering the molecular basis of biofilm development (Vlamakis et al., 2013). Formation of the extracellular matrix of *B. subtilis* biofilms is dependent on production of an exopolysaccharide synthesized by the products of the *epsA-O* operon and an amyloid-like protein component that is generated by products of the *tapA-sipW-tasA* operon (Branda et al., 2006; Romero et al., 2010). Assembly of the matrix also requires production of the bacterial hydrophobin BslA that forms a hydrophobic coat over the biofilm surface (Hobley et al., 2013; Kobayashi and Iwano, 2012; Ostrowski et al., 2011). In the laboratory, biofilms formed by *B. subtilis* manifest as robust surface-associated colonies and floating pellicles that both display a complex rugose architecture (Branda et al., 2001; Vlamakis et al., 2013).

Formation of robust colony biofilms and pellicles in *B. subtilis* is dependent on the presence of the polyamine spermidine (Burrell et al., 2010). Indeed, externally supplied spermidine can restore biofilm formation to a *B. subtilis* spermidine auxotroph. In *B. subtilis*, the triamine spermidine is formed from the diamine putrescine (Figure 1A) through transfer of an aminopropyl group to putrescine by spermidine synthase encoded by *speE* (Sekowska et al., 1998). The donor of the aminopropyl group, decarboxylated *S*-adenosylmethionine, is produced by the activity of *S*-adenosylmethionine decarboxylase, encoded by *speD* (Figure 1A) (Sekowska et al., 2000). Putrescine and, most likely, spermidine are required for biofilm formation in *Yersinia pestis*, the causative agent of bubonic plague (Patel et al., 2006; Wortham et al., 2010). However, for the cholera agent *Vibrio cholerae*, biofilm formation is dependent on biosynthesis of the unusual shorter structural analog of spermidine, norspermidine (Figure 1B), and spermidine cannot replace the function of norspermidine in biofilm formation in this species (Lee et al., 2009). Furthermore, external norspermidine is sensed by a substrate-binding protein homolog of a polyamine transporter, which then stimulates biofilm formation (Karatan et al., 2005; Karatan and Michael, 2013), whereas uptake of

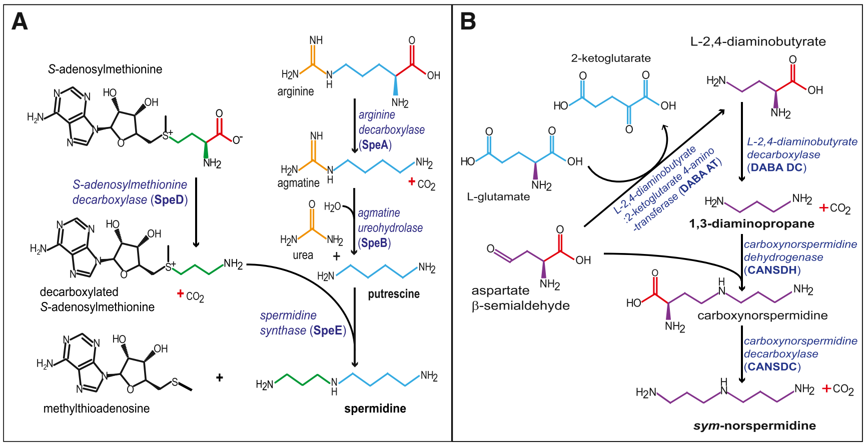

Figure 1. Spermidine and Norspermidine Biosynthetic Pathways

(A) Spermidine biosynthetic pathway of *B. subtilis* NCIB3610.

(B) Norspermidine biosynthetic pathway of *V. cholerae*.

exogenous spermidine inhibits biofilm formation (McGinnis et al., 2009). The complete pathway for norspermidine biosynthesis in *V. cholerae* (Figure 1B) has been established only recently by Lee et al. (2009) and is enzymatically distinct from the spermidine biosynthetic pathway found in *B. subtilis*. During planktonic growth, spermidine is the only detectable polyamine in *B. subtilis* (Burrell et al., 2010), whereas in *V. cholerae*, the only detectable triamine is norspermidine (Lee et al., 2009).

Recently, a study reported that *B. subtilis* synthesizes norspermidine in 8-day-old pellicle biofilms using the *V. cholerae*-type biosynthetic pathway in order to disassemble the biofilm (Kolodkin-Gal et al., 2012). The authors reported that *B. subtilis* biofilms contain 50–80 μM norspermidine and that just 25 μM exogenous norspermidine added to the growth medium prior to inoculation fully inhibits biofilm formation without inhibiting planktonic growth. It was proposed that the *B. subtilis* genes *gabT* and *yaaO* encode the norspermidine biosynthetic enzymes L-2,4-diaminobutyrate:2-ketoglutarate 4-aminotransferase (DABA AT) and carboxynorspermidine decarboxylase (CANSDC) (Figure 1B), respectively, and that mutation of either of those genes abolished norspermidine biosynthesis and prevented biofilm disassembly. The authors also proposed that norspermidine inhibited biofilm formation by binding to the exopolysaccharide. Due to the important implications of these findings for understanding biofilm physiology and the biosynthesis and function of polyamines, our laboratories each independently reexamined the key findings of Kolodkin-Gal et al. (2012). In contrast, we find that norspermidine is not synthesized by *B. subtilis*, is not naturally present in biofilms formed by *B. subtilis*, and orthologs of the *V. cholerae* norspermidine biosynthetic genes are absent from the *B. subtilis* genome. Therefore, norspermidine is unlikely to have a native role in biofilm physiology of this species, whereas the related polyamine spermidine is essential for robust biofilm formation.

RESULTS

Norspermidine Replaces the Essential Role of Spermidine in *B. subtilis* Biofilm Formation

The polyamine spermidine ($H_2N(CH_2)_3NH(CH_2)_4NH_2$) (Figure 1A) is essential for robust biofilm formation in *B. subtilis* (Burrell et al., 2010). Deletion of the spermidine biosynthetic enzymes arginine decarboxylase encoded by *speA* or *S*-adenosylmethionine decarboxylase encoded by *speD* (Figure 1A) prevents development of the highly wrinkled colony biofilm morphology of the wild-type *B. subtilis* strain NCIB3610 grown on solid polyamine-free MSgg growth medium (Figure 2A). Exogenous provision of the shorter spermidine structural analog norspermidine ($H_2N(CH_2)_3NH(CH_2)_3NH_2$) (Figure 1B) in the growth medium is more effective than exogenous spermidine in restoring the complex colony biofilm phenotype to the *speA* and *speD* spermidine-deficient mutants, whereas the longer structural analog homospermidine ($H_2N(CH_2)_4NH(CH_2)_4NH_2$) is ineffective at the same concentrations (Figure 2A). Spermidine, norspermidine, and homospermidine do not restore a normal complex colony biofilm morphology to the *tasA* and *eps* mutants, which lack the biofilm amyloid protein and exopolysaccharide, respectively. Formation of robust pellicle biofilms of *B. subtilis* NCIB3610 that develop at the liquid-air interface is also spermidine dependent (Burrell et al., 2010). The wrinkled pellicle morphology of the *B. subtilis* NCIB3610 wild-type strain is absent in the spermidine biosynthetic mutants *speA* and *speD* (Figure 2B) after 2-day incubation. Exogenous provision of just 5 μM spermidine or norspermidine to the liquid MSgg growth medium at the start of incubation restores the wrinkled pellicle morphology to the *speA* and *speD* mutants, whereas homospermidine is ineffective even at 100 μM (Figure 2B). Moreover, we noted that when exogenous norspermidine is provided to the *B. subtilis* wild-type NCIB3610 strain at higher concentrations (25 or 100 μM) for longer times (5 or 7 days), the pellicles become more wrinkled (Figure 2C). This phenotype may not be as apparent if the wild-type pellicle is already highly wrinkled. Increased wrinkling occurred with a *B. subtilis* NCIB3610 isolate maintained by us and also with an NCIB3610 isolate obtained from the Losick laboratory (referred to here as NCIB3610-H) (Figure 2C).

Higher Concentrations of Norspermidine Eventually Inhibit Planktonic Growth and Biofilm Formation

Given the essential role of spermidine in formation of *B. subtilis* NCIB3610 robust colony and pellicle biofilms and the ability of norspermidine to efficiently substitute for spermidine in this function, we were surprised by the finding of Kolodkin-Gal et al. (2012) that norspermidine at a concentration of only 25 μM prevents formation of *B. subtilis* NCIB3610 pellicle biofilms. Our two laboratories each independently assayed the effect of exogenously supplied norspermidine on pellicle formation. In contrast to the finding of Kolodkin-Gal et al. (2012) that *B. subtilis* NCIB3610 pellicle biofilm formation is inhibited by

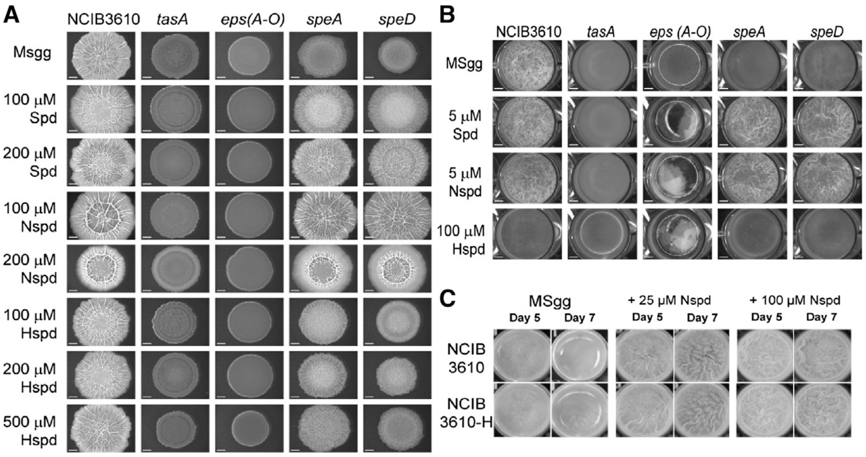

Figure 2. Norspermidine Replaces the Function of Spermidine in Biofilm Formation

(A) Complex colony biofilms of *B. subtilis* strains were grown on solid MSgg medium with or without polyamines indicated. MSgg, polyamine-free chemicallydefined growth medium; Spd, spermidine; Nspd, norspermidine; Hspd, homospermidine. Strains of *B. subtilis* included NCIB3610 (wild-type strain), *tasA* (mutant of the biofilm amyloid protein [NRS2415]), *eps(A-O)* (mutant of the exopolysaccharide biosynthetic gene cluster [NRS2450]), *speA* (mutant of arginine decarboxylase [putrescine and spermidine auxotroph, NRS3089]), and *speD* (mutant of *S*-adenosylmethionine decarboxylase [spermidine auxotroph, NRS4005]). Strains were grown at 30°C for 48 hr prior to imaging. Scale bars represent 2.5 mm.

(B) Pellicle biofilms of *B. subtilis* strains formed at the liquid-air interface on liquid growth medium after 2 days of incubation at 25°C. Strains of *B. subtilis* included NCIB3610, *eps(A-O)*, *speA*, and *speD*.

(C) Norspermidine promotes *B. subtilis* wild-type pellicle biofilm formation and rugosity. Cells of our laboratory *B. subtilis* wild-type isolate (NCIB3610) and the wild-type isolate from the Losick laboratory (NCIB3610-H) were grown for either 5 or 7 days in MSgg liquid medium with 0, 25, or 100 μM norspermidine.

25 μM norspermidine, we found that biofilm formation was not inhibited until a concentration of 250 μM norspermidine (Figure 3A). We were concerned that our laboratory isolate of *B. subtilis* NCIB3610 might differ in behavior toward exogenously provided norspermidine compared with the NCIB3610-H isolate used by Kolodkin-Gal et al. (2012). However, analysis demonstrated that the two isolates behaved very similarly, with pellicle formation being prevented by a concentration of exogenous norspermidine of between 250 and 300 μM (Figure 3A).

It was noted by Kolodkin-Gal et al. (2012) that the wispy fragments of floating material present in an exopolysaccharide mutant were unaffected by addition of norspermidine at the same concentrations that inhibited pellicle formation of *B. subtilis* NCIB3610. They postulated that norspermidine inhibited biofilm formation by interfering with the exopolysaccharide component of the biofilm matrix. In contrast, we found that at a concentration of 300 μM norspermidine, the wispy fragments of floating material in the exopolysaccharide mutant *eps (A-O)* strain no longer formed, and at 500 μM norspermidine, no residual pellicle material could be detected with the *B. subtilis* NCIB3610 *eps (A-O)* mutant (Figure 3B). These results suggested to us that norspermidine might be inhibiting the growth of planktonic cells, which in turn would lead to the biofilm defects observed. Therefore, the effect of increasing concentrations of norspermidine on planktonic growth of *B. subtilis* NCIB3610 (Figures 3C and 3E) and the *eps (A-O)* exopolysaccharide mutant (Figures 3D and 3F) was examined. A concentration of 300 μM norspermidine had a marked inhibitory effect on growth in both strains, and a concentration of 1 mM norspermidine essentially abolished growth of both strains (Figures 3C and 3D). There was little effect on planktonic growth in the presence of 1 mM spermidine (Figures 3E and 3F).

Norspermidine Is Not Present in *B. subtilis* Pellicle Biofilms

The concentration of norspermidine in 8-day-old pellicles determined by Kolodkin-Gal et al. (2012) was 50–80 μM, but the presence of spermidine was not reported. To determine whether spermidine was also present in the pellicles, we employed high-performance liquid chromatography (HPLC) with pure chemical standards to both detect and distinguish spermidine and norspermidine. Two different labeling methods were used to detect the polyamines, by attaching chemical tags that would then render them visible by fluorescence detection. The first approach used a commercial amino acid detection system (AccQ-Fluor Reagent; Waters) that derivatizes polyamines with the fluorescent AccQ-Fluor tag (Figure 4), whereas the second approach derivatized polyamines by dansylation (Figure 5).

Analysis of 3-day-old and 8-day-old *B. subtilis* NCIB3610 pellicles grown in polyamine-free MSgg medium indicated the presence of spermidine but the complete absence of norspermidine (red traces in Figures 4A and 4C). As expected, the spermidine peak was absent in residual pellicle samples derived from the spermidine biosynthetic mutants *speA* and *speD* (Figures 4D and 4F). The absence of norspermidine in 8-day-old pellicles was surprising given the finding of Kolodkin-Gal et al. (2012) that norspermidine was present at 50–80 μM. To exclude the possibility that our HPLC system was unable to detect a norspermidine concentration of 50–80 μM, we determined the limit of detection of a pure norspermidine standard added to the *B. subtilis* NCIB3610 pellicle material before extraction with trichloroacetic acid. The limit of norspermidine detection from the pellicle material was found to be well below 0.5 μM (Figure 4E). To confirm that self-produced norspermidine could be detected by our analytical system, cells of *Vibrio parahaemolyticus*, which encodes a norspermidine biosynthetic pathway highly similar to that of *V. cholerae*, were analyzed and found to contain norspermidine (Figure 4B) and some spermidine. The spermidine was likely derived from the Luria Bertani (LB) growth medium used to grow the *V. parahaemolyticus*, whereas LB medium does not contain norspermidine (McGinnis et al., 2009). As an alternative fluorescence-derivatization approach for labeling polyamines with a fluorescent chemical tag, pellicle extracts were dansylated and then analyzed by HPLC. Analysis

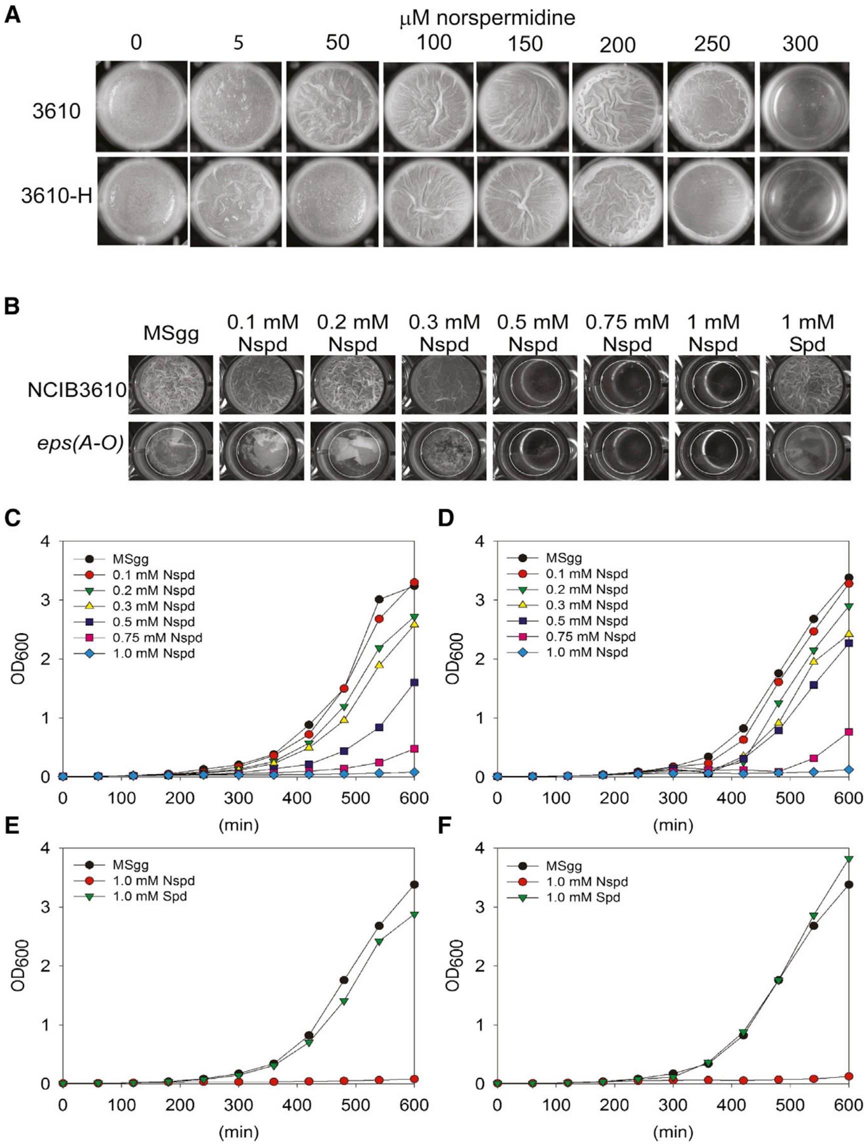

Figure 3. Higher Concentrations of Norspermidine Inhibit Growth and Pellicle Formation

(A) Effect of higher norspermidine concentrations on wild-type pellicle formation. Cells of our laboratory *B. subtilis* NCIB3610 isolate (3610) or an isolate from the Losick laboratory (3610-H) were grown for 2 days in increasing concentrations of norspermidine in liquid MSgg medium.

(B) Norspermidine inhibits the formation of the vestigial pellicle fragments of an exopolysaccharide mutant. NCIB3610, wild-type *B. subtilis*; *eps(A-O)* (NRS2450), exopolysaccharide-deficient mutant.

(C) Norspermidine inhibits the growth of *B. subtilis* NCIB3610 planktonic cells. Growth of *B. subtilis* NCIB3610 planktonic cells grown in liquid MSgg at 37°C with shaking at different concentrations of norspermidine is shown.

(D) Norspermidine inhibits the growth of *B. subtilis eps(A-O)* exopolysaccharide-deficient planktonic cells. Growth of *B. subtilis eps(A-O)* (NRS2450) planktonic cells grown in liquid MSgg at 37°C with different concentrations of norspermidine is shown.

(E) Norspermidine but not spermidine inhibits the growth of *B. subtilis* NCIB3610 planktonic cells. Growth of *B. subtilis* NCIB3610 planktonic cells grown in liquid MSgg at 37°C with shaking and no polyamine, 1 mM norspermidine (repeated from C), or 1 mM spermidine is shown.

(F) Norspermidine but not spermidine inhibits the growth of *B. subtilis eps(A-O)* exopolysaccharide-deficient planktonic cells. Growth of *B. subtilis eps(A-O)* planktonic cells grown in liquid MSgg at 37°C with shaking and no polyamine, 1 mM norspermidine (repeated from D), or 1 mM spermidine is shown.

of 1-, 3-, and 8-day-old pellicles indicated the presence of spermidine increasing in concentration from day 1 to day 3 and then declining by day 8 (Figures 5A–5C). Norspermidine was not detected at any stage of pellicle development in the NCIB3610 strain, and spermidine was absent in the *speA* strain from the corresponding stages (Figures 5D–5F). Pure norspermidine and spermidine chemical standards were easily resolved by the solvent system (Figures 5G–5I). With the dansylation method, the limit of detection for norspermidine was below 0.3 μM.

B. subtilis Lacks a Norspermidine Biosynthetic Pathway

Given our finding of a complete absence of detectable norspermidine in *B. subtilis* NCIB3610 pellicles of various ages, determined independently in each of our laboratories, and the obvious discrepancy with the findings of Kolodkin-Gal et al. (2012), we analyzed the genome of *B. subtilis* for the presence of norspermidine biosynthetic genes. The first step in the *V. cholerae* norspermidine biosynthetic pathway is conversion of aspartate β-semialdehyde to 2,4-diaminobutyrate (Ikai and Yamamoto, 1997), catalyzed by the enzyme DABA AT (EC.2.6.1.76). Kolodkin-Gal et al. (2012) asserted that norspermidine is synthesized in *B. subtilis* via the same pathway employed in *V. cholerae* (Lee et al., 2009), and they selected *gabT* as the orthologous gene encoding DABA AT. They concluded that the *gabT* mutant strain was blocked in norspermidine production. This result is surprising because previously published data demonstrated that *gabT* encodes the enzyme 4-aminobutyrate:2-ketoglutarate aminotransferase (EC.2.6.1.19) that is involved in 4-aminobutyrate (GABA) catabolism (Dover and Halpern, 1974). Another study showed that in the *B. subtilis* strain SMY, mutation of the *gabT* gene prevented utilization of GABA as a sole nitrogen source (Belitsky and Sonenshein, 2002). Here, we constructed an in-frame mutant of *gabT* in the *B. subtilis* NCIB3610 strain (Figure 6A) and confirmed a loss of growth on GABA as the sole nitrogen source (Figure 6C). Complementation of the *B. subtilis* NCIB3610 *gabT* mutant with the native *gabT* gene under control of the

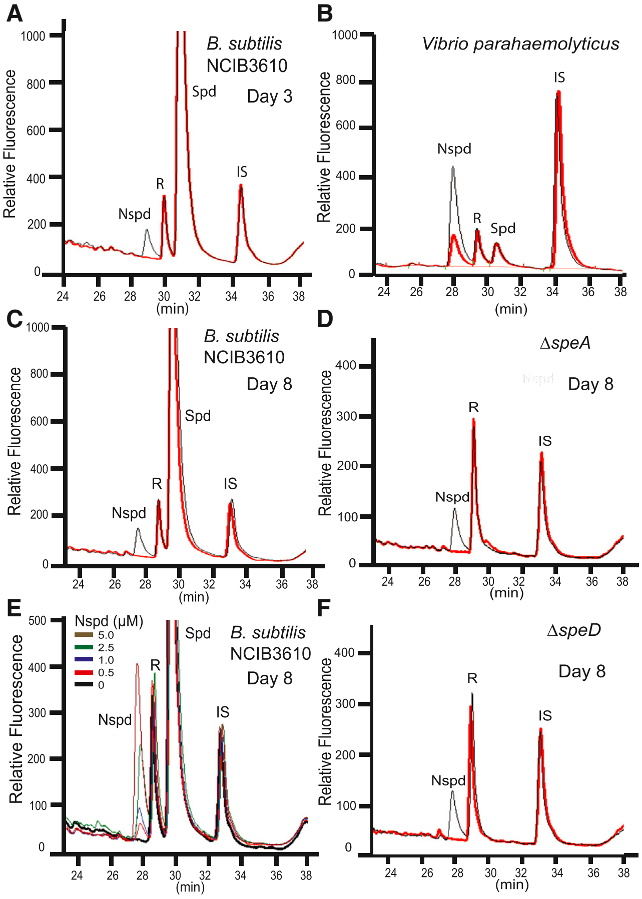

Figure 4. Norspermidine Is Not Present in *B. subtilis* NCIB3610 Pellicle Biofilms

HPLC analysis of polyamine content of pellicles. R, AccQ-Fluor reagent; IS, internal standard (1,7-diaminoheptane). Red trace indicates pellicle material, and black trace indicates pellicle material spiked with norspermidine standard before extraction with perchloric acid (except in E; see specific legend). The position of spermidine was confirmed by spiking a separate pellicle sample with pure spermidine standard (data not shown).

(A) Polyamines detected by HPLC analysis of wild-type *B. subtilis* NCIB3610 3-day-old pellicles grown in liquid MSgg medium. Representative analysis of three independent experiments is presented.

(B) Endogenous norspermidine detected by HPLC analysis of *V. parahaemolyticus* RIMD 2210633. Cells of *V. parahaemolyticus* RIMD 2210633 were grown in MLB liquid growth medium.

(C) Polyamines detected by HPLC analysis of wild-type *B. subtilis* NCIB3610 8-day-old pellicles grown in MSgg medium. Representative analysis of three independent experiments is presented.

(D) Polyamines detected by HPLC analysis of *B. subtilis speA* (NRS3089) 8-day-old pellicles grown in MSgg medium. Representative analysis of three independent experiments is shown.

(E) Limit of detection of norspermidine in 8-day-old pellicles of *B. subtilis* NCIB3610. Different concentrations of pure norspermidine standard were spiked into 8-day-old pellicles before extraction of polyamines from the pellicles with trichloroacetic acid. The wild-type trace with no added norspermidine is colored black.

(F) Polyamines detected by HPLC analysis of *B. subtilis speD* (NRS4005) 8-day-old pellicles grown in MSgg medium. Representative analysis of three independent experiments is presented.

isopropyl β-D-1-thiogalactopyranoside (IPTG)-inducible promoter ($P_{hy\text{-}spank}$) restored growth on GABA as the sole nitrogen source in the presence of IPTG (Figure 6D). Consistent with these findings, catabolism of GABA by GabT produces succinate semialdehyde, which is converted to succinate by succinate semialdehyde dehydrogenase (GabD). In *B. subtilis*, the *gabT* gene is found immediately upstream of *gabD* in a GABA catabolic operon (Figure 6A). Differences between the DABA AT reaction required for synthesis of 1,3-diaminopropane and norspermidine and the GabT reaction required for catabolism of 4-aminobutyric acid (GABA) are shown in Figure 6F. The key difference is that the product of GabT is succinate semialdehyde, which is converted by GabD to succinate for entry into the tricarboxylic acid cycle, whereas the product of DABA AT is the amino acid L-2,4-diaminobutyrate, which is then committed to polyamine biosynthesis by the action of L-2,4-diaminobutyrate decarboxylase (DABA DC).

The *B. subtilis* NCIB3610 genome contains several genes encoding proteins with some similarity to *V. cholerae* DABA AT, all of which belong to the aspartate aminotransferase family (Schneider et al., 2000). Most similar are *argD* encoding *N*-acetylornithine aminotransferase (34% aa identity), *rocD* encoding ornithine aminotransferase (30% aa identity), and as discussed above, *gabT* (30% aa identity). The Gram-positive *B. subtilis* GabT protein is more similar to GabT encoded by the Gram-negative *Acinetobacter baumannii* than to DABA AT from related *Bacillus megaterium* (Figure S1A available online). Furthermore, the *B. subtilis*, *B. megaterium*, and *A. baumannii gabT* open reading frame (ORF) is found clustered with a *gabD* ORF, whereas the DABA AT-encoding ORF from *A. baumannii*, *B. megaterium*, and *V. cholerae* is found adjacent or fused to a DABA DC-encoding ORF (Figure S1B). Thus, confirmed enzymatic activity (Belitsky and Sonenshein, 2002), sequence similarity, and operon context demonstrate that the *B. subtilis* GabT is involved in GABA catabolism and not biosynthesis of norspermidine. The next step in the *V. cholerae* norspermidine biosynthetic pathway is the conversion of L-2,4-diaminobutyrate produced by DABA AT to 1,3-diaminopropane, accomplished by DABA DC (EC.4.1.1.68). Sequence analysis indicates that there are no homologs of DABA DC in any sequenced *B. subtilis* genome except for a distant homolog (YP_005559741) encoded in *B. subtilis* subspecies Natto BEST195; however, this genome does not encode DABA AT. In conclusion, *B. subtilis* does not encode the biosynthetic pathway for the norspermidine precursor 1,3-diaminopropane.

After conversion of L-2,4-diaminobutyrate to 1,3-diaminopropane, the next step in norspermidine production in *V. cholerae* is conversion of 1,3-diaminopropane to carboxynorspermidine by the enzyme carboxynorspermidine dehydrogenase (CANSDH) (Figure 1B). We find that no homologous gene is present in any sequenced *B. subtilis* genome. Carboxynorspermidine is then converted to norspermidine by CANSDC. Kolodkin-Gal et al. (2012) selected the *yaaO* gene as the *B. subtilis* ortholog of CANSDC and found that a *yaaO* mutant strain produced a longer-lasting pellicle. However, our protein sequence comparison analysis (Figure 7) indicates that YaaO exhibits negligible sequence similarity with the biochemically characterized CANSDC proteins from *V. cholerae* and *Campylobacter jejuni*.

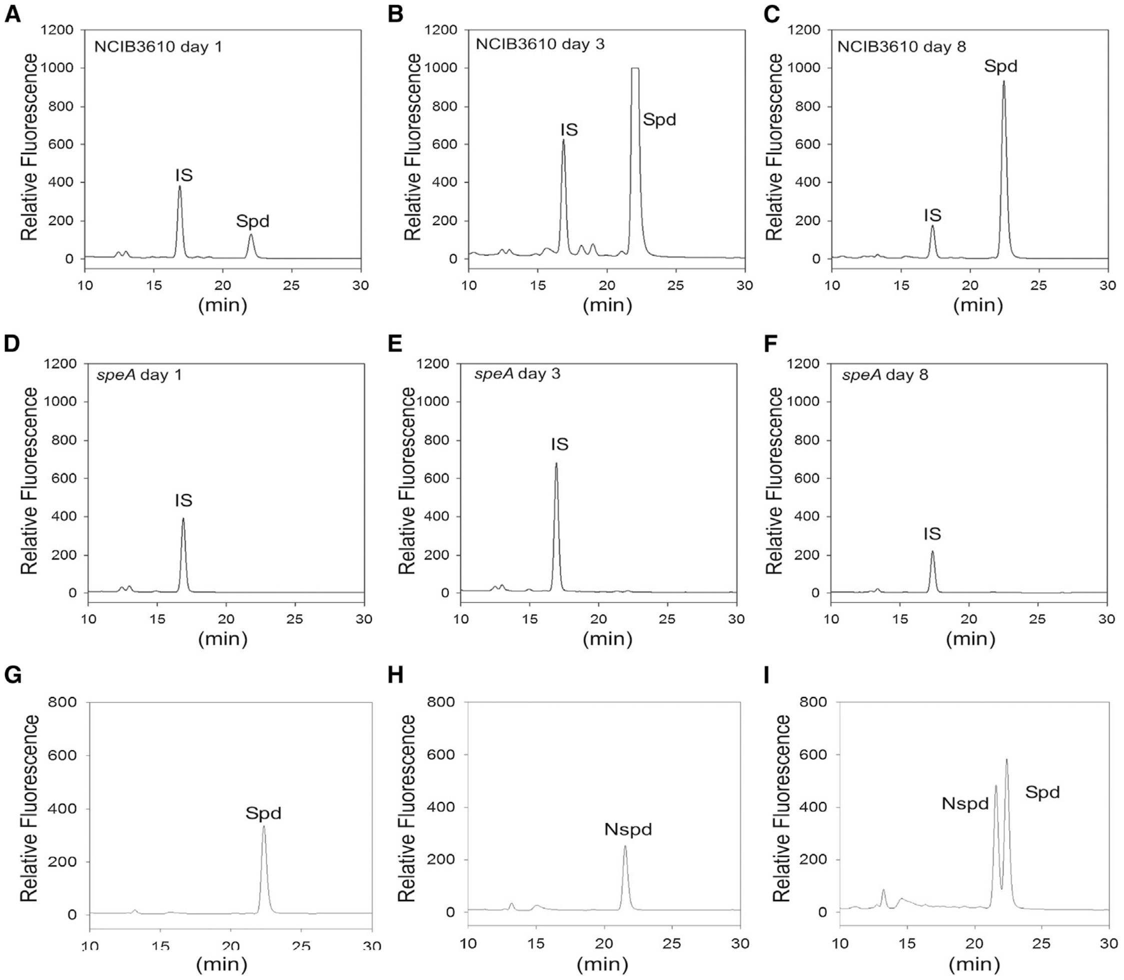

Figure 5. Spermidine Is Present at All Stages of *B. subtilis* Pellicle Biofilm Development

Polyamines were detected by HPLC after derivatization with dansyl chloride.

(A–C) Polyamine content in wild-type *B. subtilis* NCIB3610 pellicle biofilms at days 1, 3, and 8 of development, respectively.

(D–F) Polyamine content in pellicles of the *B. subtilis speA* polyamine auxotrophic mutant at days 1, 3, and 8 of development, respectively.

(G–I) Pure spermidine, norspermidine, and combined spermidine and norspermidine chemical standards, respectively.

Instead, YaaO is a homolog of the *B. subtilis* SpeA (YP_007533417) biosynthetic arginine decarboxylase (34.9% aa identity in 467 aa overlap) and the *E. coli* AdiA acid-inducible arginine decarboxylase protein (Burrell et al., 2010) and is therefore from a different protein fold to CANSDC (Deng et al., 2010). In short, genes encoding CANSDH and CANSDC are absent from all *B. subtilis* genomes. An endogenous plasmid, pBS32 is present in *B. subtilis* NCIB3610 (Konkol et al., 2013), but DABA AT, DABA DC, CANSDH, and CANSDC are not encoded on this plasmid. Finally, we resequenced the genomes of the two isolates of *B. subtilis* used in our experiments (NCIB3610 and NCIB3610-H) and compared them with the reference genome of *B. subtilis* 168 (Srivatsan et al., 2008). There are 29 SNPs unique to the isolate of *B. subtilis* NCIB3610 from our laboratory and 28 SNPs unique to the *B. subtilis* NCIB3610-H isolate (Table S1). None of the SNPs in either NCIB3610 isolate affects any genes related to polyamine metabolism.

DISCUSSION

The biosynthesis of norspermidine in *V. cholerae* is achieved by four different enzymatic steps that have been biochemically characterized and the corresponding *V. cholerae* genes identified (Ikai and Yamamoto, 1994, 1997; Lee et al., 2009; Nakao et al., 1991; Yamamoto et al., 1986). Based on our comparative genomic analyses described above, it is clear that there are no homologs of DABA DC, CANSDH, or CANSDC in *B. subtilis* NCIB3610. Distant homologs of DABA AT are present, but they have known functions and are not involved in norspermidine biosynthesis: *argD* encoding *N*-acetylornithine aminotransferase (Mountain et al., 1986), *rocD* encoding ornithine aminotransferase (Gardan et al., 1995), and *gabT* encoding GABA aminotransferase (Belitsky and Sonenshein, 2002). Thus, the claim by Kolodkin-Gal et al. (2012) that the genes *gabT* and *yaaO* encode orthologs of *V. cholerae* DABA AT and CANSDC is

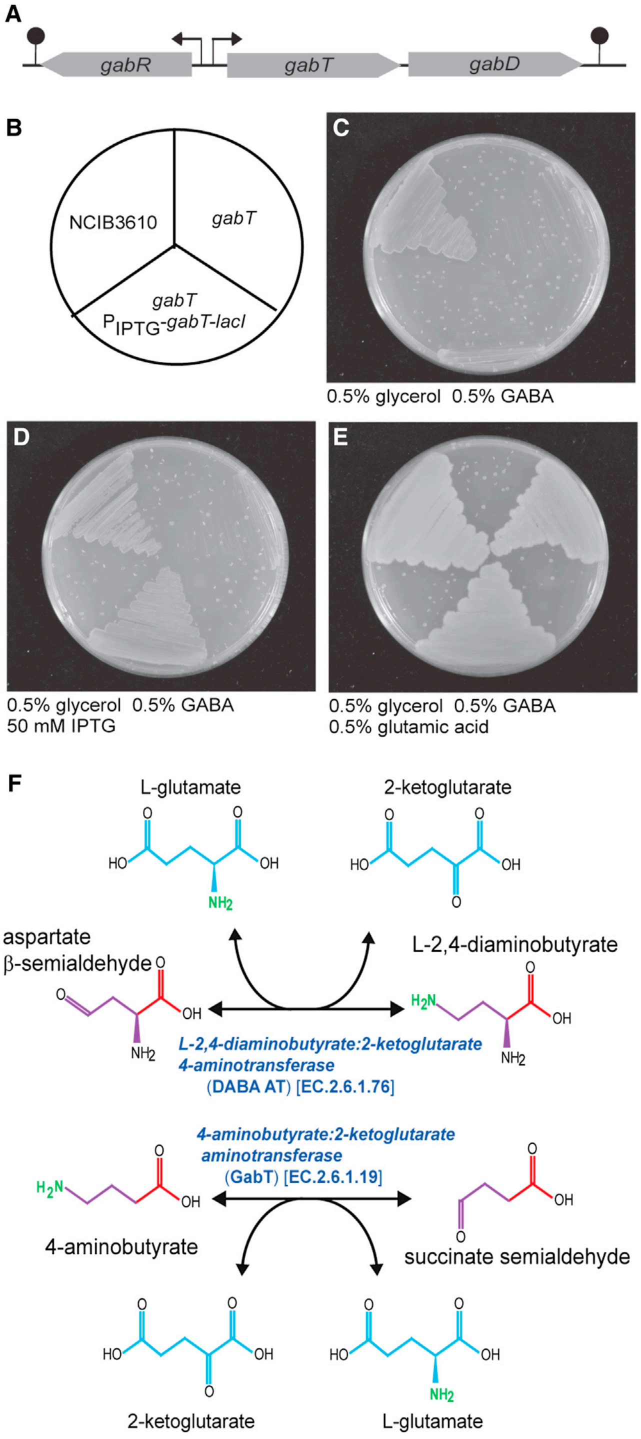

Figure 6. GabT Is Involved in GABA Catabolism and Not Biosynthesis of Norspermidine

(A) Schematic of the *B. subtilis* GABA catabolic operon. The transcription factor-encoding *gabR* ORF is transcribed divergently from the *gabT* and *gabD* ORFs. Arrows and filled circles represent transcriptional promoters and terminators, respectively.

(B) Schematic of the arrangement of *B. subtilis* strains grown on solid MSgg growth medium plates. Top-left view shows the wild-type *B. subtilis* NCIB3610, top-right view shows *B. subtilis gabT* mutant (NRS4007), and bottom view shows *B. subtilis gabT* mutant expressing an IPTG-inducible wild-type *gabT* ORF from the heterologous *amyE* site on the chromosome (NRS4104).

(C) The *B. subtilis* strains detailed above grown on MS medium containing 0.5% GABA as the sole nitrogen source and 0.5% glycerol as the carbon source.

(D) Strains detailed above grown on MS medium containing 0.5% GABA as the sole nitrogen source, with induction of the complimenting *gabT* gene by 50 mM IPTG.

(E) Strains detailed above grown on MS medium containing both 0.5% GABA and 0.5% glutamic acid as the nitrogen source.

(F) The DABA AT and GabT reactions.

erroneous. Not only does YaaO exhibit no homology to CANSDC (Figure 7), but *B. subtilis gabT* has been shown previously to be essential for GABA catabolism (Belitsky and Sonenshein, 2002). We cannot explain the discrepancies between our findings and those of Kolodkin-Gal et al. (2012), although we note that the mass spectrometric analysis of norspermidine presented by those authors was that of a pure norspermidine chemical standard from Sigma-Aldrich and not of a biological sample (Figure S1 of Kolodkin-Gal et al., 2012). Indeed, the only data presented that examined the presence of norspermidine within a biological sample are the liquid chromatography-mass spectrometry (LC-MS) analysis of pellicle material presented in their Figure 1C, which presents only retention times and not mass analysis. Moreover, it should be noted that this figure does not include a control pellicle sample spiked with a pure norspermidine standard to indicate where the norspermidine elutes after extraction from the biological matrix. In the absence of any other supporting evidence, it is not clear why it was concluded by Kolodkin-Gal et al. (2012) that norspermidine was present in the pellicle material. It is also unclear why spermidine was not detected because it is abundant in pellicles at all developmental stages and elutes from an LC column very close to norspermidine.

To ascertain the level of norspermidine in the *B. subtilis* 8-day-old pellicles, our two laboratories independently attempted to detect norspermidine at various stages of pellicle development. At physiological pH, the amine groups of polyamines such as norspermidine are fully protonated and bind strongly to polyanionic molecules. To extract all noncovalently bound polyamines from pellicles, we used an aggressive extraction procedure using trichloroacetic acid (CCl_3COOH). We note that Kolodkin-Gal et al. (2012) used PBS to extract polyamines, which may account for the lack of detection of spermidine. Nevertheless, we did not detect any norspermidine in 8-day-old pellicles or in pellicles at any stage, although our limit of detection was below 0.5 μM, i.e., less than 1% of the concentration reported by Kolodkin-Gal et al. (2012). In contrast, we found that spermidine was abundant at all stages of pellicle development but decreased with increasing age of the pellicle (Figures 4 and 5). Furthermore, we found that norspermidine detection from cells of *V. parahaemolyticus*, which possesses the norspermidine biosynthetic pathway, was facile (Figure 4B).

We determined that the concentration of norspermidine that inhibited pellicle formation (250 μM) was ten times higher than that reported by Kolodkin-Gal et al. (2012). Those authors suggested that norspermidine triggers biofilm disassembly by

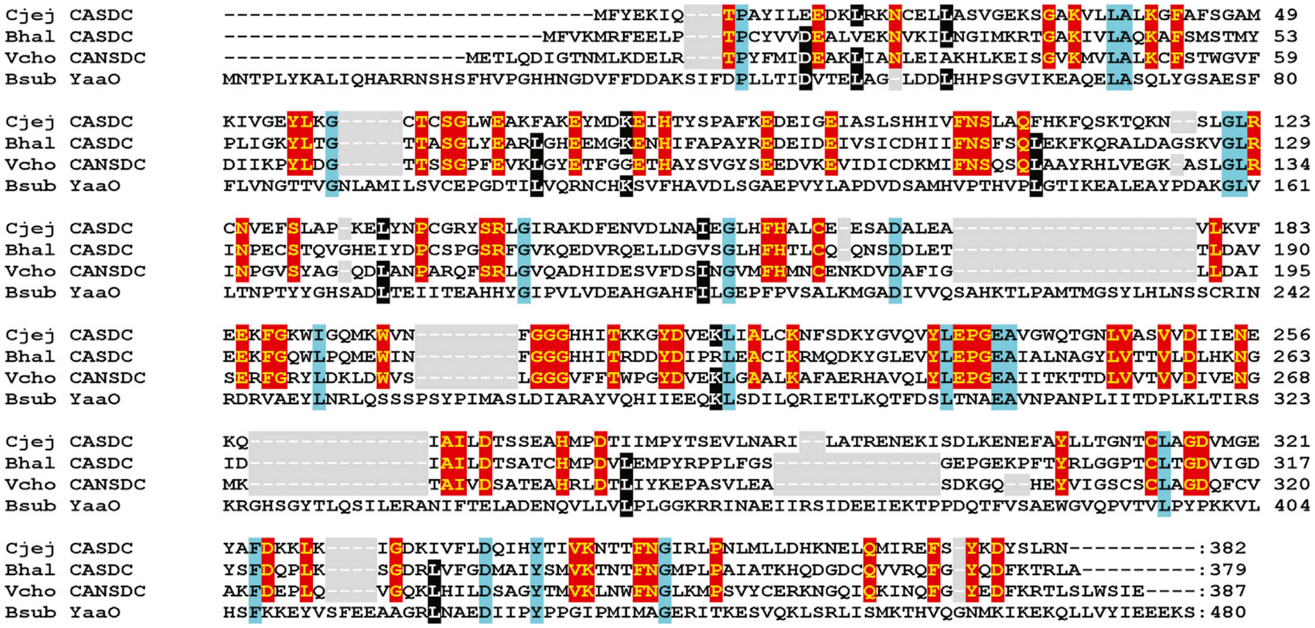

Figure 7. YaaO Is Not a Homolog of CANSDC

Alignment (CLUSTALW) is shown for the amino acid sequences of the *C. jejuni* CANSDC (GenBank WP_002855819 for Cjej CASDC), *B. halodurans* CANSDC (GenBank NP_244826) homolog (Bhal CASDC), *V. cholerae* CANSDC (GenBank NP_231262 for Vcho CANSDC), and the *B. subtilis* YaaO protein (GenBank NP_387908 for Bsub YaaO). Gaps in the sequence alignment are highlighted in gray, red highlights the identity within the CANSDC/CASDC family of proteins, blue highlights the identity in all four sequences, and black highlights the identity in three out of four sequences regardless of family. See also Figure S1.

targeting exopolysaccharide. This idea was based on their observation that the residual pellicles of wild-type cells that developed in the presence of norspermidine resembled the wispy fragments of floating material seen for a mutant blocked in exopolysaccharide production. However, in Figure S2B of Kolodkin-Gal et al. (2012), the provision of 25 μM exogenous norspermidine in the growth medium appears to have significantly reduced the amount of residual wispy pellicle material in the exopolysaccharide mutant, a result not commented upon by the authors. We found that the wispy floating fragments of the exopolysaccharide mutant did not develop when grown in concentrations of norspermidine that fully inhibited pellicle formation in the wild-type strain (Figure 3B). When *B. subtilis* NCIB3610 wild-type cells were grown planktonically by Kolodkin-Gal et al. (2012) (see Figure S4 in this reference) in liquid MSgg with exogenous norspermidine present at a concentration 4-fold above the minimum inhibitory concentration (MIC) for pellicle formation determined by the authors (4 × 25 μM), they observed no inhibition of growth. In contrast, when we grew planktonic wild-type cells in a concentration of norspermidine 4-fold above our observed MIC for pellicle formation (4 × 250 μM), planktonic growth was abolished for both wild-type (Figure 3C) and exopolysaccharide mutant (Figure 3D) cells. We found that planktonic growth of both wild-type and exopolysaccharide mutant cells is equally sensitive to lower concentrations of exogenous norspermidine (Figures 3A and 3B). The in vitro effects of norspermidine on the hydrodynamic radii of exopolysaccharide reported by Kolodkin-Gal et al. (2012) (see Figure 4 in this reference) were observed using 0.75 mM norspermidine, a concentration 30-fold higher than their reported MIC for biofilm development. Our results indicate that exogenous norspermidine does not act through the targeting of exopolysaccharide; instead, norspermidine inhibits growth per se, which is likely to be the predominant inhibitory factor preventing pellicle development.

In conclusion, we find that *B. subtilis* NCIB3610 does not encode orthologs of the *V. cholerae* norspermidine biosynthetic pathway. Pellicle biofilms of *B. subtilis* do not contain natively produced norspermidine at any stage of development, and exogenous norspermidine is not inhibitory to pellicle development until a concentration 10-fold higher than reported by Kolodkin-Gal et al. (2012). We find that exogenous norspermidine inhibits planktonic growth and pellicle development in an exopolysaccharide-independent manner. Our data demonstrate that norspermidine is not a self-produced trigger for biofilm disassembly in *B. subtilis* and reconfirm the essential role of spermidine for formation of robust biofilms, a role that can be replaced more effectively at the same concentration by the nonnative polyamine norspermidine.

EXPERIMENTAL PROCEDURES

Bacterial Strains and Growth Conditions

Strains of *B. subtilis* used in this study are described in Table S2. All *E. coli* and *B. subtilis* strains were routinely grown in LB liquid medium (10 g NaCl, 5 g yeast extract, and 10 g tryptone/l) or on solid medium plates containing 1.5% Select Agar (Invitrogen) at 37°C. *V. parahaemolyticus* RIMD 2210633 (a kind gift of K. Orth, University of Texas Southwestern Medical Center) was grown in modified LB medium (MLB; tryptone 10 g, yeast extract 5 g, and

NaCl 30 g in 1 liter) at 30°C. For growth in polyamine-free liquid medium, strains were grown in MSgg medium (5 mM potassium phosphate and 100 mM MOPS at pH 7.0 supplemented with 2 mM $MgCl_2$, 700 μM $CaCl_2$, 50 μM $MnCl_2$, 50 μM $FeCl_3$, 1 μM $ZnCl_2$, 2 μM thiamine, 0.5% glycerol, and 0.5% glutamate) (Branda et al., 2001) or on solid MSgg plates (solidified with 1.5% Select Agar). When appropriate, antibiotics were used at the following concentrations: 100 μg/ml ampicillin, 100 μg/ml spectinomycin, 12.5 μg/ml tetracycline, 0.5 μg/ml erythromycin, and 12.5 μg/ml lincomycin. Spermidine and *sym*-norspermidine were obtained from Sigma-Aldrich, and *sym*-homospermidine was a kind gift from Patrick Woster (Medical University of South Carolina).

Strain Construction

All *B. subtilis* strains, plasmids, and primers used and constructed in this study are listed in Tables S2, S3, and S4. For the construction and maintenance of plasmids, *E. coli* strain MC1061 (*F'lacIQ lacZM15 Tn10* (*tet*)) was used. Derivatives of *B. subtilis* 168 were generated by transformation of competent cells with plasmids using standard protocols of Harwood and Cutting (1990). For introduction of DNA into *B. subtilis* strain NCIB3610, SPP1 phage transductions were conducted as described previously by Verhamme et al. (2007).

Strains containing in-frame gene deletions of *speD* and *gabT* were constructed using a modified version of a previously published method (Kiley and Stanley-Wall, 2010). The upstream region of *speD* was PCR amplified with primers NSW1500 and NSW1501, and the downstream region with 1502 and 1503. The two purified products were linked by further PCR using the external primers 1500 and 1503. This product was cut with BamHI and HindIII and ligated into pUC19 cut with the same restriction enzymes, creating the vector pNW1106, and the insert was then released by digestion with BamHI and BglII and ligated into pMAD, creating vector pNW1111. The construct for deletion of *gabT* was created in a similar way: upstream DNA was amplified with NSW1508 and NSW1509 and downstream with NSW1510 and NSW1511; the two products were then joined by PCR. Purified PCR product was cut with XmaI and HindIII and ligated into pUC19, creating pNW1107, and the insert was then released with XmaI and BglII and ligated into pMAD, creating the vector pNW1110. Strains NRS4005 (NCIB3610 Δ*speD*) and NRS4007 (NCIB3610 Δ*gabT*) were created by integration and curing of pNW1111 and pNW1110, respectively, in NCIB 3610 as previously described (Kiley and Stanley-Wall, 2010).

To introduce *gabT* under the control of the IPTG-inducible $P_{hy\text{-}spank}$ promoter at the nonessential *amyE* locus, the coding region of *gabT* with its ribosome-binding site was amplified from NCIB3610 genomic DNA with the primers NSW1524 and NSW1525. The purified product was cut with HindIII and SphI, then ligated into pDR111 cut with the same enzymes, creating the vector pNW1114.

Colony and Pellicle Biofilm Formation

Strains of *B. subtilis* were grown overnight on MSgg plates at 37°C. Single colonies were inoculated into 3 ml MSgg and grown with shaking at 37°C for 6 hr. To set up complex colonies, 10 μl of cells was spotted on MSgg plates and incubated for 48 hr at 30°C before imaging. For pellicle analysis, pellicles were formed in either 2 or 10 ml of MSgg broth (with 2 or 10 μl of cells as inoculum, respectively) and were grown at 25°C for the stated length of time.

Planktonic Growth Analysis

B. subtilis strains were streaked out to single colonies on an MSgg plate and grown overnight at 37°C. Single colonies were inoculated into 25 ml of MSgg broth and grown overnight at 37°C with shaking. Cells were diluted to an optical density at 600 nm (OD_{600nm}) of 0.01 in 25 ml of fresh MSgg broth and incubated at 37°C with shaking at 200 rpm. Polyamines were added to the growth media at the final concentrations indicated immediately prior to inoculation. Planktonic growth was measured by OD_{600nm} readings.

Growth on 4-Aminobutyric Acid as Sole Nitrogen Source

Strains of *B. subtilis* were streaked out to single colonies on an MSgg plate and grown overnight at 37°C. Single colonies were then streaked onto MS plates (5 mM potassium phosphate and 100 mM MOPS at pH 7.0 supplemented with 2 mM MgCl2, 700 μM CaCl2, 50 μM MnCl2, 50 μM FeCl3, 1 μM ZnCl2, and 2 μM thiamine) with 0.5% glycerol as a carbon source, and with either 0.5% glutamic acid or 0.5% 4-aminobutyric acid (GABA) or both as a nitrogen source. For induction of the *gabT*-complemented strain, 50 μM IPTG was added to the growth medium. Plates were grown again at 37°C overnight before being imaged.

Preparation of Pellicle Material for Derivatization of Polyamines with AccQ-Fluor Reagent

Pellicles of *B. subtilis* NCIB3610 and derivative strains were grown in 10 ml of liquid MSgg in 6-well plates (Greiner Bio-One). The 10 ml of pellicle material and spent medium was transferred to a 15 ml Falcon tube and centrifuged to pellet the pellicle material including both cells and matrix. The pellet was resuspended with 0.25 ml H_20, and then 40% trichloroacetic acid was added to a final concentration of 10% and incubated on ice for 30 min. This pellicle suspension was subjected to three cycles of freeze/thawing. After centrifugation (18,000 × *g*, 5 min, 4°C), the resulting supernatant was used for immediate analysis or stored at −20°C until needed. The 1,7-diaminoheptane internal standard and pure polyamine standards were added to the pellicles immediately before extraction of the pellicle material by trichloroacetic acid.

Derivatization of Samples with AccQ-Fluor Reagent and HPLC Analysis

Analysis of trichloroacetic acid-extracted polyamines was performed by precolumn derivatization with AccQ-Fluor reagent labeling kit (Waters; catalog No. WAT052880). For each sample (5 μl), derivatization was carried out in a total volume of 100 μl AccQ-fluor borate buffer and heated to 55°C for 10 min. Polyamines labeled with the AccQ-Fluor reagent were separated by HPLC with a hydrolysate amino acid analysis column (AccQ-Tag column, 60 Å, 4 μm, 3.9 × 150 mm; Waters) on a Beckman Coulter System Gold with fluorescence detection (excitation, 248 nm; emission, 398 nm [Prostar]). The solvent system consisted of solvent A (520 mM sodium acetic acid, 17 mM triethylamine, and 0.01% sodium azide [pH 4.65]) and solvent B (60% acetonitrile, 0.01% acetone at a flow rate of 1.0 ml/min). Elution was performed using a linear gradient of buffer B: 2–6 min, 0%–20%; 6–11 min, 20%; 11–27 min, 20%–50%; 27–34 min, 50%; 34–37 min, 100%; and 40–50 min, 0%.

Calculation of the Limit of Detection of Norspermidine in Pellicle Material

Eight-day-old pellicle material grown in 10 ml of MSgg medium in 6-well plates was centrifuged and resuspended in H_2O to a volume of 350 μl. One of several norspermidine stock solutions of different concentrations was added (9.6 μl), along with 15 μl of 3 mM 1,7-diaminoheptane to the resuspended pellicle material, which included cells and pellicle matrix, mixed and then extracted with 117 μl of trichloroacetic acid and made up to a final volume of 500 μl. The apparent concentration of the norspermidine spike in the sample was taken as the concentration of norspermidine in the 500 μl final volume of extracted pellicle suspension. After three cycles of freeze/thawing, the suspension was centrifuged and then 5 μl of the supernatant was added to 75 μl of borate buffer and 20 μl of AccQ-Fluor reagent. After the labeling reaction, 20 μl of this was used for HPLC analysis.

Pellicle Biofilm Sample Preparation for HPLC Analysis

Pellicles formed in 10 ml of liquid MSgg were set up as described above. Samples were taken for analysis at various days after inoculation. The pellicle material and liquid media were both transferred to a centrifuge tube and centrifuged at 4,000 × *g* for 10 min. The pellet representing all pellicular material, both cells and biofilm matrix were analyzed by HPLC as described below.

Preparation of Pellicle Material for Dansylation of Polyamines

Pellicles formed in 10 ml of liquid MSgg were set up as described above. Samples were taken for analysis at various days after inoculation. The pellicle material and liquid medium were both transferred to a 15 ml Falcon tube and centrifuged at 3,750 rpm for 10 min. Supernatant was removed, and the pellicle pellet was resuspended in 0.25 ml H_2O for 1-day and 8-day pellicles and 0.5 ml for 3-day pellicles. After resuspension, 15 μl of 0.4 mM 1,7-diaminoheptane

(the internal standard) was added to the samples before addition of the trichloroacetic acid. An equal volume (to the H_2O used for resuspension) of trichloroacetic acid (made in 10 mM HCl) was added to the resuspended pellicle samples. The solution was kept on ice for 30 min to precipitate protein, which was pelleted by centrifugation, and the supernatant was transferred to another tube. This supernatant was extracted five times with water-saturated ethyl acetate (polyamines remain in the bottom layer; the top layer was discarded). The liquid was removed by freeze-drying overnight, and the solid material was reconstituted with 50 μl 10 mM HCl.

Derivatization of Samples with Dansyl Chloride and HPLC Analysis

Fresh dansyl chloride (5-dimethylaminonapthalene-1-sulphonyl chloride) solution was prepared as 10 mg/ml in redistilled acetone, wrapped in foil, and kept on ice (Kabra et al., 1986). A total of 200 μl saturated Na_2CO_3 (pH rises to approximately 10) and 200 μl of dansyl chloride solution was added to 50 μl of the pellicle sample. After mixing, the solution was heated to 70°C for 10 min, cooled to room temperature, and then 100 μl 25% (w/v) L-proline was added to mop up excess dansyl chloride. The top acetone layer was retained for HPLC analysis using ion-paired reverse-phase HPLC on an Ultrasphere C18 column using a Dionex Ultimate 3000 instrument fitted with a Dionex RF-2000 fluorometer (set at 340 nm excitation and 515 nm emission). The solvent system consisted of solvent A (10 mM sodium phosphate monobasic phosphate buffer [$NaH_2PO_4.H_2O$] [pH 4.2]) and solvent B (acetonitrile). Elution was performed at a flow rate of 2.0 ml/min, the column equilibrated in 40% solvent B for 8 min, then a linear gradient of solvent B: 0–25 min, 40%–65%; 25–35 min, 65%–90%; and 35–42 min, 90%. See the Extended Experimental Procedures for more information.

SUPPLEMENTAL INFORMATION

Supplemental Information includes Extended Experimental Procedures, four tables, and one figure and can be found with this article online at http://dx.doi.org/10.1016/j.cell.2014.01.012.

AUTHOR CONTRIBUTIONS

Experiments were designed, performed, and analyzed by N.R.S.-W., A.H.F., L.H., S.W., S.H.K., Y.M., and A.J.M. L.H. and N.R.S.-W. constructed strains, and L.H., N.R.S.-W., and Y.M. performed biofilm and cell growth assays. S.H.K., S.W., and A.H.F. optimized and performed polyamine analysis on samples prepared by L.H. and S.H.K. L.H. prepared samples for whole-genome SNP analysis. SNP identification was by L.H. and N.R.S.-W. with further bioinformatics analysis by N.R.S.-W., A.H.F., and A.J.M. The paper was written and edited by A.J.M., A.H.F., N.R.S.-W., and L.H.

ACKNOWLEDGMENTS

We thank David Mangelsdorf for helpful discussions and http://www.sarahjcgillespie.com for design work on the graphical abstract. This work was supported by grant BB/I019464/1 from the Biotechnology and Biological Sciences Research Council, UK (to N.R.S.-W.), by grant 079838 from the Wellcome Trust (to A.H.F.), and by a High Impact/High Risk Award from UT Southwestern Medical Center (to A.J.M.). Genome sequencing and analysis were performed at the Genomic Sequencing Unit, University of Dundee, which is funded by the Wellcome Trust Strategic Grant (098439/Z/12/(Z)). We thank Prof. Richard Losick for providing strain NCIB3610-H.

Received: October 16, 2013
Revised: December 19, 2013
Accepted: January 7, 2014
Published: February 13, 2014

REFERENCES

Belitsky, B.R., and Sonenshein, A.L. (2002). GabR, a member of a novel protein family, regulates the utilization of gamma-aminobutyrate in *Bacillus subtilis*. Mol. Microbiol. *45*, 569–583.

Branda, S.S., González-Pastor, J.E., Ben-Yehuda, S., Losick, R., and Kolter, R. (2001). Fruiting body formation by *Bacillus subtilis*. Proc. Natl. Acad. Sci. USA *98*, 11621–11626.

Branda, S.S., Vik, S., Friedman, L., and Kolter, R. (2005). Biofilms: the matrix revisited. Trends Microbiol. *13*, 20–26.

Branda, S.S., Chu, F., Kearns, D.B., Losick, R., and Kolter, R. (2006). A major protein component of the *Bacillus subtilis* biofilm matrix. Mol. Microbiol. *59*, 1229–1238.

Burrell, M., Hanfrey, C.C., Murray, E.J., Stanley-Wall, N.R., and Michael, A.J. (2010). Evolution and multiplicity of arginine decarboxylases in polyamine biosynthesis and essential role in *Bacillus subtilis* biofilm formation. J. Biol. Chem. *285*, 39224–39238.

Costerton, J.W., Lewandowski, Z., Caldwell, D.E., Korber, D.R., and Lappin-Scott, H.M. (1995). Microbial biofilms. Annu. Rev. Microbiol. *49*, 711–745.

Davey, M.E., and O'Toole, G.A. (2000). Microbial biofilms: from ecology to molecular genetics. Microbiol. Mol. Biol. Rev. *64*, 847–867.

Deng, X., Lee, J., Michael, A.J., Tomchick, D.R., Goldsmith, E.J., and Phillips, M.A. (2010). Evolution of substrate specificity within a diverse family of beta/alpha-barrel-fold basic amino acid decarboxylases: X-ray structure determination of enzymes with specificity for L-arginine and carboxynorspermidine. J. Biol. Chem. *285*, 25708–25719.

Dover, S., and Halpern, Y.S. (1974). Genetic analysis of the gamma-aminobutyrate utilization pathway in *Escherichia coli* K-12. J. Bacteriol. *117*, 494–501.

Flemming, H.C., and Wingender, J. (2010). The biofilm matrix. Nat. Rev. Microbiol. *8*, 623–633.

Gardan, R., Rapoport, G., and Débarbouillé, M. (1995). Expression of the rocDEF operon involved in arginine catabolism in *Bacillus subtilis*. J. Mol. Biol. *249*, 843–856.

Harwood, C.R., and Cutting, S.M. (1990). Molecular Biological Methods for *Bacillus* (Chichester: John Wiley & Sons).

Hobley, L., Ostrowski, A., Rao, F.V., Bromley, K.M., Porter, M., Prescott, A.R., MacPhee, C.E., van Aalten, D.M., and Stanley-Wall, N.R. (2013). BslA is a self-assembling bacterial hydrophobin that coats the *Bacillus subtilis* biofilm. Proc. Natl. Acad. Sci. USA *110*, 13600–13605.

Ikai, H., and Yamamoto, S. (1994). Cloning and expression in *Escherichia coli* of the gene encoding a novel L-2,4-diaminobutyrate decarboxylase of *Acinetobacter baumannii*. FEMS Microbiol. Lett. *124*, 225–228.

Ikai, H., and Yamamoto, S. (1997). Identification and analysis of a gene encoding L-2,4-diaminobutyrate:2-ketoglutarate 4-aminotransferase involved in the 1,3-diaminopropane production pathway in *Acinetobacter baumannii*. J. Bacteriol. *179*, 5118–5125.

Joo, H.S., and Otto, M. (2012). Molecular basis of in vivo biofilm formation by bacterial pathogens. Chem. Biol. *19*, 1503–1513.

Kabra, P.M., Lee, H.K., Lubich, W.P., and Marton, L.J. (1986). Solid-phase extraction and determination of dansyl derivatives of unconjugated and acetylated polyamines by reversed-phase liquid chromatography: improved separation systems for polyamines in cerebrospinal fluid, urine and tissue. J. Chromatogr. A *380*, 19–32.

Karatan, E., and Michael, A.J. (2013). A wider role for polyamines in biofilm formation. Biotechnol. Lett. *35*, 1715–1717.

Karatan, E., Duncan, T.R., and Watnick, P.I. (2005). NspS, a predicted polyamine sensor, mediates activation of *Vibrio cholerae* biofilm formation by norspermidine. J. Bacteriol. *187*, 7434–7443.

Kiley, T.B., and Stanley-Wall, N.R. (2010). Post-translational control of *Bacillus subtilis* biofilm formation mediated by tyrosine phosphorylation. Mol. Microbiol. *78*, 947–963.

Kobayashi, K., and Iwano, M. (2012). BslA(YuaB) forms a hydrophobic layer on the surface of *Bacillus subtilis* biofilms. Mol. Microbiol. *85*, 51–66.

Kolodkin-Gal, I., Cao, S., Chai, L., Böttcher, T., Kolter, R., Clardy, J., and Losick, R. (2012). A self-produced trigger for biofilm disassembly that targets exopolysaccharide. Cell *149*, 684–692.

Konkol, M.A., Blair, K.M., and Kearns, D.B. (2013). Plasmid-encoded ComI inhibits competence in the ancestral 3610 strain of *Bacillus subtilis*. J. Bacteriol. *195*, 4085–4093.

Lee, J., Sperandio, V., Frantz, D.E., Longgood, J., Camilli, A., Phillips, M.A., and Michael, A.J. (2009). An alternative polyamine biosynthetic pathway is widespread in bacteria and essential for biofilm formation in *Vibrio cholerae*. J. Biol. Chem. *284*, 9899–9907.

McGinnis, M.W., Parker, Z.M., Walter, N.E., Rutkovsky, A.C., Cartaya-Marin, C., and Karatan, E. (2009). Spermidine regulates *Vibrio cholerae* biofilm formation via transport and signaling pathways. FEMS Microbiol. Lett. *299*, 166–174.

Mountain, A., McChesney, J., Smith, M.C., and Baumberg, S. (1986). Gene sequence encoding early enzymes of arginine synthesis within a cluster in *Bacillus subtilis*, as revealed by cloning in *Escherichia coli*. J. Bacteriol. *165*, 1026–1028.

Nakao, H., Shinoda, S., and Yamamoto, S. (1991). Purification and some properties of carboxynorspermidine synthase participating in a novel biosynthetic pathway for norspermidine in *Vibrio alginolyticus*. J. Gen. Microbiol. *137*, 1737–1742.

Ostrowski, A., Mehert, A., Prescott, A., Kiley, T.B., and Stanley-Wall, N.R. (2011). YuaB functions synergistically with the exopolysaccharide and TasA amyloid fibers to allow biofilm formation by *Bacillus subtilis*. J. Bacteriol. *193*, 4821–4831.

Patel, C.N., Wortham, B.W., Lines, J.L., Fetherston, J.D., Perry, R.D., and Oliveira, M.A. (2006). Polyamines are essential for the formation of plague biofilm. J. Bacteriol. *188*, 2355–2363.

Romero, D., Aguilar, C., Losick, R., and Kolter, R. (2010). Amyloid fibers provide structural integrity to *Bacillus subtilis* biofilms. Proc. Natl. Acad. Sci. USA *107*, 2230–2234.

Schneider, G., Käck, H., and Lindqvist, Y. (2000). The manifold of vitamin B6 dependent enzymes. Structure *8*, R1–R6.

Sekowska, A., Bertin, P., and Danchin, A. (1998). Characterization of polyamine synthesis pathway in *Bacillus subtilis* 168. Mol. Microbiol. *29*, 851–858.

Sekowska, A., Coppée, J.Y., Le Caer, J.P., Martin-Verstraete, I., and Danchin, A. (2000). S-adenosylmethionine decarboxylase of *Bacillus subtilis* is closely related to archaebacterial counterparts. Mol. Microbiol. *36*, 1135–1147.

Srivatsan, A., Han, Y., Peng, J., Tehranchi, A.K., Gibbs, R., Wang, J.D., and Chen, R. (2008). High-precision, whole-genome sequencing of laboratory strains facilitates genetic studies. PLoS Genet. *4*, e1000139.

Verhamme, D.T., Kiley, T.B., and Stanley-Wall, N.R. (2007). DegU co-ordinates multicellular behaviour exhibited by *Bacillus subtilis*. Mol. Microbiol. *65*, 554–568.

Vlamakis, H., Chai, Y., Beauregard, P., Losick, R., and Kolter, R. (2013). Sticking together: building a biofilm the *Bacillus subtilis* way. Nat. Rev. Microbiol. *11*, 157–168.

Wortham, B.W., Oliveira, M.A., Fetherston, J.D., and Perry, R.D. (2010). Polyamines are required for the expression of key Hms proteins important for *Yersinia pestis* biofilm formation. Environ. Microbiol. *12*, 2034–2047.

Yamamoto, S., Hamanaka, K., Suemoto, Y., Ono, B., and Shinoda, S. (1986). Evidence for the presence of a novel biosynthetic pathway for norspermidine in *Vibrio*. Can. J. Microbiol. *32*, 99–103.

Supplemental Information

EXTENDED EXPERIMENTAL PROCEDURES

SNP Comparisons of NCIB3610 and NCIB3610-H

Genomic DNA from planktonically grown cells of each strain was extracted using the DNeasy Blood and Tissue Kit (QIAGEN) using the protocol for Gram-positive bacteria. The DNA was quantified using the QuBit 2.0 DNA kit, and 1 μg of DNA was sheared into 300 bp fragments using a Covaris M220 Focused Ultrasonicator. Paired end libraries were generated using the Illumina TruSeq DNA sample preparation guide, and sequenced using the Illumina MiSeq Reagent kit v2 on the Illumina MiSeq platform. The sequence data was analyzed using MiSeq Reporter, and alignment to the reference genome (*B. subtilis* 168, GenBank Accession NZ_CM000487, version NZ_ZM000487.1 GI:223666304, and *B. subtilis* NCIB3610 plasmid pAS32, GenBank Accessions ABQL01000016.1 and ABQL01000023.1) was done using BWA and variant calling to identify SNPs was performed using GATK.

SUPPLEMENTAL REFERENCES

Arnaud, M., Chastanet, A., and Débarbouillé, M. (2004). New vector for efficient allelic replacement in naturally nontransformable, low-GC-content, gram-positive bacteria. Appl. Environ. Microbiol. *70*, 6887–6891.

Britton, R.A., Eichenberger, P., Gonzalez-Pastor, J.E., Fawcett, P., Monson, R., Losick, R., and Grossman, A.D. (2002). Genome-wide analysis of the stationary-phase sigma factor (sigma-H) regulon of *Bacillus subtilis*. J. Bacteriol. *184*, 4881–4890.

Yanisch-Perron, C., Vieira, J., and Messing, J. (1985). Improved M13 phage cloning vectors and host strains: nucleotide sequences of the M13mp18 and pUC19 vectors. Gene *33*, 103–119.

A

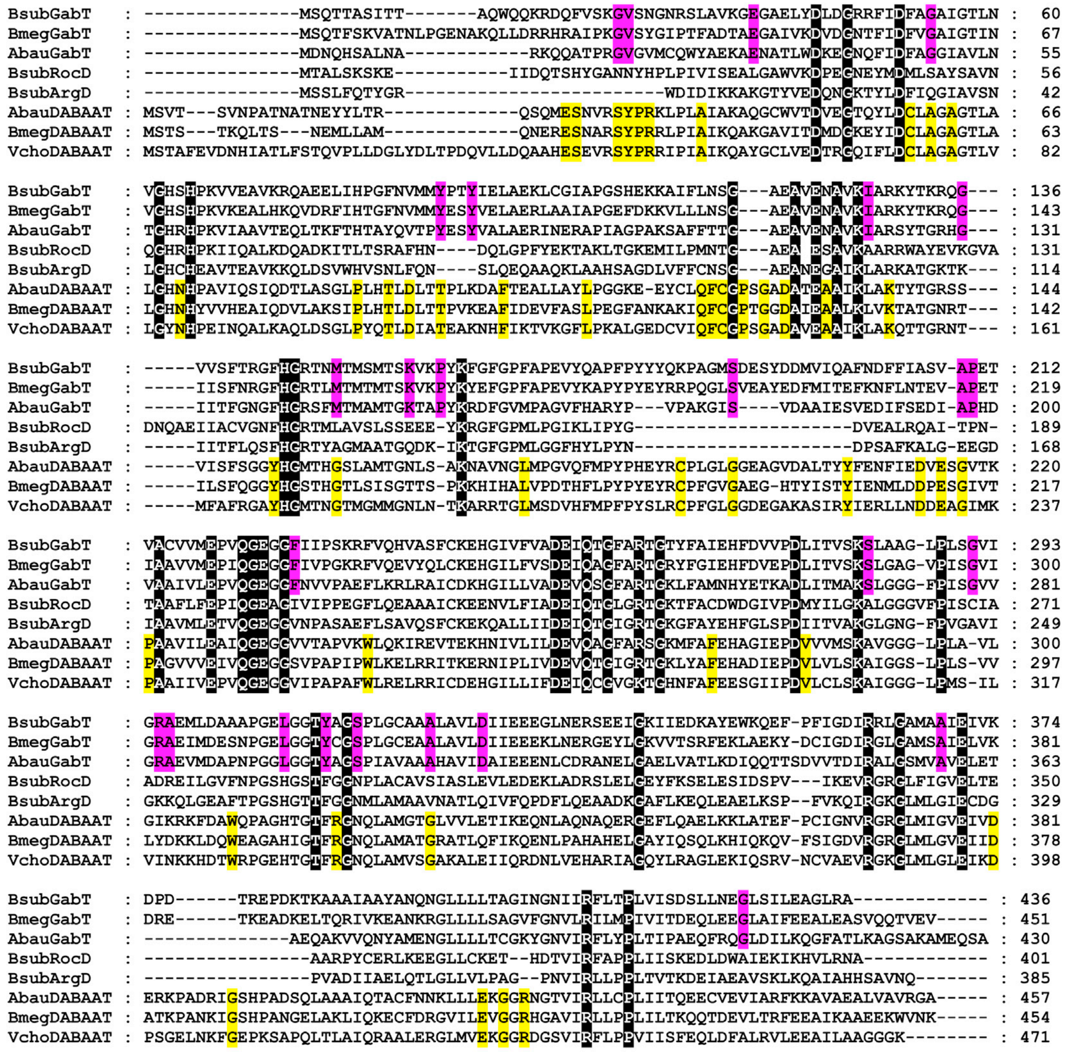

B

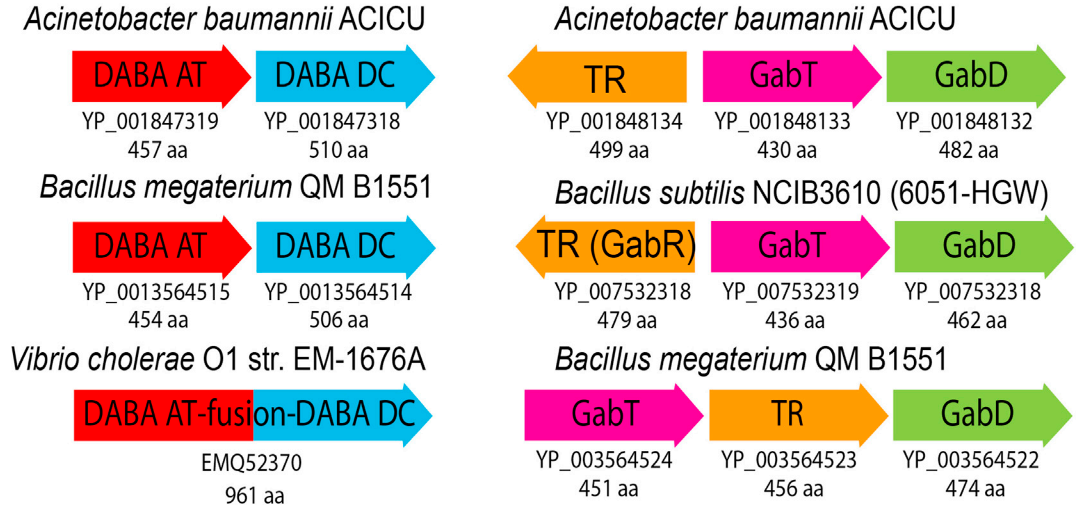

(legend on next page)

Figure S1. Sequence Alignment and Gene Clusters of GabT and DABA AT Proteins, Related to Figure 7

(A) Sequence alignment of GabT, DABA AT, RocD and ArgD proteins. An alignment of 4-aminobutyrate:2-ketoglutarate aminotransferase (GabT) proteins from *Bacillus subtilis*, *Bacillus megaterium* and *Acinetobacter baumannii*; L-2,4-diaminobutyrate:2-ketoglutarate 4-aminotransferase (DABA AT) proteins from *Vibrio cholerae*, *A. baumannii*, and *B. megaterium*; ornithine aminotransferase (RocD) from *B. subtilis*; and *N*-acetylornithine aminotransferase (ArgD) from *B. subtilis*. Proteins: BsubGabT, *B. subtilis* NCIB3610 [6051-HGW] GabT (YP_007532319); BmegGabT, *B. megaterium* QM B1551 GabT (YP_003564524); AbauGabT, *A. baumannii* ACICU GabT (YP_001848133); BsubRocD, *B. subtilis* NCIB3610 RocD (YP_007536035); BsubArgD, *B. subtilis* NCIB3610 ArgD (YP_007533057); AbauDABAAT, *A. baumannii* ACICU DABA AT (YP_001847319); BmegDABAAT, *B. megaterium* QM B1551 DABA AT (YP_003564515); VchoDABAAT, *V. cholerae* O1 str. EM-1676A DABA AT (EMQ52370). Identical amino acid positions conserved only in the Gram-positive (*B. subtilis* and *B. megaterium*) and Gram-negative (*A. baumannii*) GabT proteins are highlighted in red. Identical amino acids positions conserved only in the Gram-positive (*B. megaterium*) and Gram-negative (*V. cholerae* and *A. baumannii*) DABA AT proteins are highlighted in yellow. Identical amino acid positions conserved in all DABA AT, GabT, RocD and ArgD proteins are highlighted in black.

(B) Gene clusters containing DABA AT or GabT ORFs. DABA AT and GabT ORFs from *Acinetobacter baumannii*, *Bacillus megaterium*, *Bacillus subtilis* and *Vibrio cholerae*. The GenBank protein accession numbers are provided beneath each ORF as well as the size of the ORF (in amino acids). DABA DC, 2,4-diaminobutyrate decarboxylase; TR, transcription regulator; GabD, succinate semialdehyde dehydrogenase.
